# Models of survivorship care in patients with head and neck cancer in regional, rural, and remote areas: a systematic review

**DOI:** 10.1007/s11764-024-01643-x

**Published:** 2024-07-20

**Authors:** Poorva Pradhan, Ashleigh R. Sharman, Carsten E. Palme, Michael S. Elliott, Jonathan R. Clark, Rebecca L. Venchiarutti

**Affiliations:** 1 Department of Head and Neck Surgery, Chris O’Brien Lifehouse, Camperdown, New South Wales Australia; 2 Central Clinical School, Faculty of Medicine and Health, University of Sydney, Camperdown, New South Wales Australia; 3 Royal Prince Alfred Institute of Academic Surgery, Sydney Local Health District, Camperdown, New South Wales Australia; 4 Sydney School of Public Health, The University of Sydney, Sydney, New South Wales Australia

**Keywords:** Neoplasm, Oncology, Head and neck cancer, Rural health, Models of care, Cancer survivorship

## Abstract

**Purpose:**

Rural people with head and neck cancers (HNC) are likely to experience poorer health outcomes due to limited access to health services, so many benefit from models of care that account for rurality. The aim of this review was to synthesise literature on models of care in this population.

**Methods:**

Studies were identified using seven databases: PubMed, PsycINFO, Scopus, Embase, CINAHL, Medline, and Web of Science. Studies that tested or reported a model of care in rural HNC survivors were included. Data on characteristics and outcomes of the models were synthesised according to the domains in the Cancer Survivorship Care Quality Framework, and study quality was appraised.

**Results:**

Seventeen articles were included. Eight were randomised controlled trials (seven with a control group and one single-arm study). Three models were delivered online, nine via telehealth, and five in-person. Majority were led by nurses and allied health specialists and most addressed management of physical (*n* = 9) and psychosocial effects (*n* = 6), while only a few assessed implementation outcomes such as cost-effectiveness. None evaluated the management of chronic health conditions.

**Conclusion:**

Positive outcomes were reported for domains of survivorship care that were measured; however, further evaluation of models of care for rural people with HNC is needed to assess effectiveness across all domains of care.

**Implications for Cancer Survivors:**

Rural cancer survivors are a diverse population with unique needs. Alternative models of care such as shared care, or models personalised to the individual, could be considered to reduce disparities in access to care and outcomes.

**Supplementary information:**

The online version contains supplementary material available at 10.1007/s11764-024-01643-x.

## Introduction

In 2022, more than 19 million people were diagnosed with cancer globally, with cancer continuing to represent a significant contribution to disease burden and premature death [1]. Incidence of head and neck cancer (HNC), which refers to an anatomically diverse group of cancers arising in the upper aerodigestive tract, varies widely around the world, with the highest in Melanesia and Southeast Asia [2]. Substantial progress has been made in early detection and improved treatment for HNC, particularly for human papillomavirus-related cancers [3]. As a result, there are growing number of people achieving remission and living beyond their diagnosis [4].

Owing to these increasing survival rates, individuals with HNC often experience long-term challenges [5], which can stem from the tumour itself, as well as the complex and morbid treatments needed to achieve a cure [6]. These include physical changes (e.g. fatigue, pain, disfigurement), psychological effects (e.g. anxiety, depression, fear of recurrence), and social consequences (e.g. relationship changes, social isolation) [5]. For survivors of HNC, body image issues are particularly pronounced due to visible alterations from treatment, such as scarring and loss of facial function. These changes can profoundly affect self-concept and identity, leading to diminished self-worth and overall well-being. Sebri et al. [7] and James [8] highlight the importance of addressing these issues to improve quality of life and psychological health. In recognition of these challenges, there has been an increased focus on developing different models of care to address the long-term aspects of cancer survivorship in recent years [9]. A model of care is broadly defined as the ‘way the health-care services are delivered’ [10]. The overarching goal of these models of care is to provide tailored and comprehensive follow-up cancer care [11].

In many countries, oncology specialist-led models of survivorship care predominate; however, workforce shortages mean other models of care need to be considered to meet the needs of the rising number of cancer survivors [9]. Alternative models include nurse-led, general practitioner (GP)-led, shared care (shared responsibility between a healthcare provider and patients and/or caregivers), or patient self-management [12]. GP-led models of care, for instance, offer significant benefits including personalised and continuous care due to the pre-existing patient-GP relationship. It enhances accessibility and convenience, particularly for rural patients. Research shows that GP-led care provides non-inferior clinical outcomes in detecting cancer recurrence and managing health-related quality of life [9]. Nurse-led and shared care approaches have also been shown to increase patient satisfaction by providing more convenience in terms of shorter travel and waiting times, and economic benefits compared to specialist-led care [13–16]. For instance, cancer nurses are trained in addressing symptom management and health promotion needs [9].

The availability and wider implementation of models of survivorship care in rural settings continue to be an important challenge [17, 18]. The drivers of successful delivery of a model of care can be different depending on where it is implemented. Patients and clinicians in rural settings encounter distinct challenges in accessing and delivering cancer care compared to those in metropolitan settings [19], especially for oncology specialist-led models of care [20]. Singh and Goebel [20] argue that individuals in rural areas face challenges in accessing cutting-edge digital technologies, making the delivery of online-based models of care difficult. Models of care that require rural dwellers to travel long distances for follow-up care increase the financial burden on individuals who often pay out of pocket for transport and accommodation to metropolitan centres [20, 21]. This financial burden can lead to rural dwellers opting out of treatment or follow-up care [22, 23], which could explain rural-urban disparities in cancer outcomes observed in many geographically dispersed countries [24–26]. Consequently, health needs including supportive care needs are left unaddressed [18].

Various models of care are designed for individuals with cancer [12] and for those with HNC [27, 28]. However, there is insufficient information as to which models of care are implemented and effective in rural areas for people with HNC. The aim of this review is to synthesise the literature on models of care for HNC survivors in rural areas and to determine how effectively they address survivorship needs.

## Methodology

This systematic review was conducted in accordance with the PRISMA 2020 statement [29]. The protocol of this review was prospectively registered with PROSPERO (CRD42022380912).

### Search strategy

A search of PubMed, PsycINFO, Scopus, Embase, CINAHL, Medline, and Web of Science databases was conducted from inception to June 2024. There was no restriction on publication period or language. The search strategy used a combination of keywords related to HNC types, location, and model of care type, and combined using ‘AND’ function to yield results (Supplementary File S1). In addition to these database searches, Google Scholar and reference lists of included studies were searched to locate additional studies.

### Eligibility criteria

Studies were eligible if they met the following inclusion criteria as per PICO (population, intervention, comparison, and outcome) approach:

- Population: Studies with adult participants (aged ≥ 18 years) who had been diagnosed and treated for HNC and who lived in rural areas.
- Intervention: Studies that described or evaluated a model of care as an intervention addressing survivorship issues. There were no limitations on the type and duration of intervention.
- Comparator(s)/control: The comparator was only applicable to interventional studies that evaluated a model of care. We included studies with or without a comparator/control group (e.g. standard of care).
- Outcome(s): Studies were included if they provided results on effectiveness outcomes relevant to the survivorship needs (e.g. psychosocial outcomes including quality of life, depression, anxiety, or distress).

### Screening and data extraction

Titles and abstracts were screened for inclusion by three authors (PP, RV, and AS). Full texts of the included citations after the title and abstract screening were performed by the same authors. Any disagreements were resolved by discussions between PP and RV. Data extraction was independently conducted by two authors (PP and AS). This extraction was checked for accuracy by a third author (RV). The following information was extracted from eligible studies: study characteristics (country of origin, study aim, and design), participant demographic characteristics (age, gender distribution, and tumour site), model of care information (number of arms: single or double, delivery mode, and outcomes reported on targeted domain within the quality of care framework), type of model of care (for example, nurse-led, primary care practitioner or GP-led, oncology specialist-led), and any other quantitative data on effectiveness outcomes (such as effect sizes, if available). The authors of included papers were contacted for missing information.

### Quality appraisal

We adopted the Joanna Briggs Institute checklists [30] corresponding to each study design to evaluate the risk of bias in the included articles. One author (RV) independently conducted study quality assessment, and 10% of these assessments were checked by another author (PP) for accuracy. Each item on the checklist was scored as follows: yes = 2, N/A = 1, and no or unclear = 0. A total score for each study was calculated and was divided by the number of items in the checklist to obtain a percentage in terms of study quality. Hence, quality was assessed as follows: high (≥ 80%), medium (60–79%), and low (< 60%).

### Data synthesis

Data were synthesised according to the Cancer Survivorship Care Quality Framework [31] and narratively reported. This framework comprises three domains related to cancer survivorship care: (i) cancer and its treatment, (ii) general healthcare, and (iii) contextual domains of healthcare delivery. For instance, when a model of care reported on outcomes such as distress or anxiety, these were categorised as ‘Surveillance and Management of Psychosocial Effects’. Likewise, outcomes related to physical symptoms such as pain and swallowing, the study was classified under ‘Surveillance and Management of Physical Effects’. We also reported data on implementation outcomes (for example, acceptability, efficacy, feasibility, and satisfaction) based on the Conceptual Framework for Implementation Outcomes [32]. For example, outcomes such as ‘cost’ were mapped under ‘implementation outcomes’ and ‘satisfaction’ under ‘client outcomes’.

The data extraction template was piloted by two authors (PP and RV) to ensure consistency, and thereafter, 50% of studies were independently extracted by two authors (PP and AS). Discrepancies were resolved through discussions among authors (PP, RV, and AS).

## Results

### Study selection

**Fig. 1:**
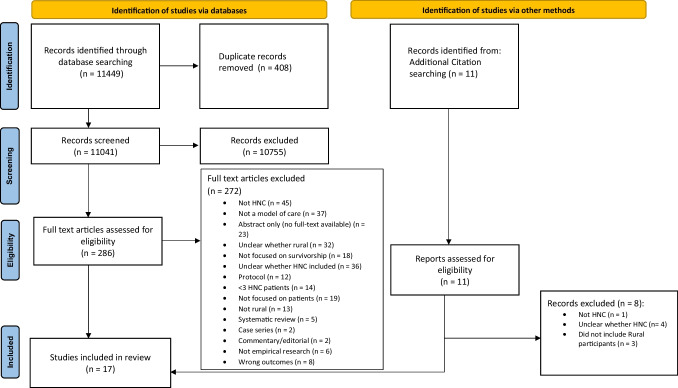
The PRISMA 2020 statement: an updated guideline for reporting systematic reviews. From: Page MJ, McKenzie JE, Bossuyt PM, Boutron I, Hoffmann TC, Mulrow CD, et al. BMJ 2021;372:n71. 10.1136/bmj.n71. For more information, visit: http://www.prisma-statement.org/

The study identification process is illustrated in Fig. 1. The initial keyword-based search yielded 9488 articles. Following the screening of titles and abstracts, 10,755 articles were excluded. The remaining articles (*n* = 286) underwent further assessment for eligibility based on their full texts, resulting in the exclusion of 272 articles. Additionally, a supplementary search was conducted based on a reference list of the included articles, and three studies were identified to be eligible. A total of 17 studies were included in the review.

### Characteristics of included studies

Study characteristics are presented in Table 1. Most were conducted in the United States (US) (*n* = 7), six in Australia, two in Canada, and one each in Italy and India. Most of the studies adopted a purposive sampling approach to enrol participants such as recruiting from rural oncology practices or hospitals. Of the 14 included studies, eight adopted a randomised trial methodology, with the majority having two intervention arms (*n* = 7). These studies were conducted across a variety of settings, with the most common being hospitals (*n* = 8) and oncology practice/clinics (*n* = 3). Only one study recruited patients from multiple settings such as community, healthcare, and regional cancer centres. Most models of care were delivered via telehealth (*n* = 9); five were delivered in-person and three online. Of the 17 studies, six were led by nurses, five entailed a self-guided care (whereby cancer survivors managed their own care), and three were led by allied health professionals, all involving speech pathologists. Two studies utilised a shared-care model. Importantly, none of the studies reported the formal criteria used to assess participants’ rurality, and the majority stated that participants were sampled based on their distance to major metro cities or cancer centres.

**Table 1:** Study characteristics table

Author, country	Assessment of rurality	Data collection period	Delivery mode (online/in-person) and setting (hospital/community)	Type of model of care	HNC sample size	Mean age	Cancer site/sub-site	Survivorship phase
Beatty et al. [40], Australia	Not reported	Not reported	Online, hospital	Self-guided	6	Not reported	Specific sites not reported for HNC patients	Post diagnosis and treatment
Bernacchi et al. [47], USA	Time required to reach the tertiary cancer centre	Not reported	Telemedicine-delivered video intervention, academic	Nurse-led	15	60.7	Not reported	Post-treatment
Burns et al. [34], Australia	Purposive sample from tertiary and regional hospital	5 months	Telehealth video, hospital	Speech pathologist-led	18	Range 42–80 years	Not reported	Pre-treatment and post-treatment
Burns et al. [35], Australia	Distance from major metro city (Brisbane)	July 2013 and October 2015	Teleconsultation, hospital	Allied health-led/speech path	TMOC, 47; SMOC, 44	TMOC, 64 years; SMOC, 65 years	Mostly laryngeal	Pre-treatment and post-treatment
Burns et al. [43], Australia	Distance from major metro city (Brisbane)	July 2013 and October 2015	Teleconsultation, hospital	Allied health-led/speech path	TMOC, 47; SMOC, 44	TMOC, 64 years; SMOC, 65 years	Mostly laryngeal	Pre-treatment and post-treatment
Cascella et al. [36], Italy	The maximum covered distance (from the cancer centre, Campania Region) was 555.22 km	March 2021 to September 2022	Telehealth, hospital	Shared-care	14	Not reported	Specific sites not reported for HNC patients	Treatment and post-treatment
DeGuzman et al. [39], USA	Not reported	Not reported	Telemedicine-delivered video interventions, academic	Nurse-led	Not reported	59.9 years	Thyroid 8; oral cavity 6; pharynx 4; other 3	Post-treatment
Dirkse et al. [41], Canada	Not reported	July 2016–March 2017	1. Self-guided: remote 2. Technician guided: through email contact or telephonic sessions; academic	Self-management	4	Not reported	Not reported	Post-treatment
Kang et al. [45], Australia	Treatment location centre (Townsville)	January 2015 to April 2019	In-person, hospital	Oncology-led	5	Not reported	Not reported	Treatment
Kroenke et al. [33], USA	Purposive sample from community-based urban and rural oncology practices	August 2008–August 2009	By phone, oncology practice	Nurse-led	16; intervention *n* = 13; control *n* = 3	Not reported	Tongue *n* = 2; tonsillar *n* = 7; nasopharyngeal *n* = 3; other *n* = 4	Treatment, post-treatment
Peterson et al. [48], USA	Purposive sample from cancer centre with urban and rural patients	Two non-consecutive 5-day periods, separated by 2 weeks of non-usage	Online, home and academic	Patient-led	31	56 years; range = 23–78 years	Not reported	Post-treatment
Risendal et al. [49], USA	Purposive sample from state-wide community cancer centres and hospital-based cancer programmes	August 2011–January 2013	In-person, variety of settings (48% community, 30% healthcare, 22% regional/community cancer centre)	CTA trainer-led (including researchers, community members, cancer survivors); self-management	5	Not reported	Not reported	Post-treatment
Sandell et al. [46], Australia	Purposive sample from regional and rural centres	Not reported	In-person, hospital	Shared-care	54	Not reported	Not reported	Post-treatment
Silva-Nash et al. [42], USA	Purposive sample within a rural state	December 2016–October 2020	In-person, clinic	Nurse-led	570	64.1 years	Multiple head and neck sites	Post-treatment, 1-year follow-up
Sivabalan and Upasani. [38], India	Purposive sample from a rural hospital	Not reported	In-person, hospital	Nurse-led	Experimental *n* = 19; control *n* = 17	Not reported	Not reported	Treatment (chemotherapy)
Yoo et al. [44], USA	Purposive sample from community-based urban and rural oncology practices	12 months	By phone, clinic	Physician and nurse-led	16; intervention *n* = 13; control *n* = 3	Not reported	Tongue *n* = 2; tonsillar *n* = 7; nasopharyngeal *n* = 3; other *n* = 4	Treatment
Zernicke et al. [37], Canada	Purposive sample from provincial cancer registry	March 2011 to August 2012	Online, home	Instructor and self-led	HNC = 4 (6.5%)	Overall sample = 57.56 years	Not reported	Post-treatment

### Study population

The total number of participants with HNC in the included studies was 976, with an age range of 23–80 years. The average age of the HNC-specific sample was not reported in most studies (*n* = 11). Majority of studies recruited patients with multiple cancer types and did not specify the HNC site (*n* = 9). Among the 17 studies, eight reported on specific HNC subsites, with the most common being oral cancers (including tongue and tonsil; *n* = 4). Laryngeal and nasopharyngeal cancers were each included in three studies and one study each for pharyngeal and parotid tumours. Most studies recruited patients across multiple survivorship phases: post-treatment (*n* = 14), during treatment (*n* = 6), and three studies before treatment. None included patients from palliative or end of life care. Eligible participants in the included studies (*n* = 7) had received multiple treatments, while in one study, recruited participants were treated with chemotherapy only. Seven studies did not report on the type of treatment patients had received.

### Quality appraisal

The quality appraisal scores are displayed in Table 1 and Supplementary Table 1. Among the included studies, seven were of high methodological quality, seven were of low quality, and three were classified as medium quality. The most common sources of bias across interventional studies (studies that assessed a model of care as an intervention) were the absence of a control group and unclear or lack of blinding of both participants and researchers to treatment assignment. Additional reasons included the lack of measurement of outcome variable across multiple time points and the unclear or lack of report on confounding factors.

### Effects of models of care on survivorship outcomes

Models of cancer care were grouped according to the outcomes they reported into domains as specified by Nekhlyudov et al.’s [31] framework. No studies addressed surveillance of chronic conditions, and health promotion and disease prevention domains and are thus not reported (see Table 2).

**Table 2:** Detailed study outcomes

Author, country	Study aims	No. of arms (single-arm or 2) and intervention description	Outcomes measures/domains of care	Main findings	Quality of cancer survivorship care framework domains	Study quality/risk of bias
Beatty et al. [40] Australia	To examine the efficacy of FMY in reducing cancer-related distress and improving quality of life compared to an online attention control	Two: 1. FMY: 6 week/6 module (or 1 module/week) iCBT programme comprising (1) psycho-education, (2) cognitive behaviour therapy-based strategies (worksheets, quizzes, relaxation/meditation exercises), and (3) survivor testimonials in video and written formats 2. Attention control: 6 module information-only website	1. Cancer distress Secondary outcomes: 1. General distress (depression, anxiety and stress) 2. QoL 3. Coping 4. Health service utilisation	Intervention group reported higher emotional functioning on QoL and accessed fewer health services. Both groups reported reductions in distress. No other significant differences were observed	1. Surveillance and management of psychosocial effects	High
Bernacchi et al. [47] USA	To understand rural survivors’ experiences of participating in a nurse-led telehealth visit designed to address cancer-related distress	Single-arm quasi-experimental design	TSUQ	Findings indicated high satisfaction with the nurse-patient relationship through telehealth	Implementation outcome: satisfaction	Low
Burns et al. [34], Australia	To explore the feasibility of providing access to specialist speech pathology services via telehealth for head and neck cancer patients	Single-arm. Weekly telehealth clinic lasting 4 h provided from tertiary hospital setting	Feasibility; acceptability; satisfaction	The model of care was found to be feasible and acceptable, and patients were overall satisfied with the delivery. All cases were successfully managed via telehealth	1. Surveillance and management of physical effects 2. Implementation outcome: cost-effectiveness and satisfaction	Medium
Burns et al. [35], Australia	To assess the economic impact of a new synchronous telepractice service delivering speech pathology intervention to nonmetropolitan head and neck cancer patients	Two: 1. TMOC: an online consultation between the metro cancer centre and regional centres, utilising a videoconferencing unit with audiovisual recordings of the clinical area of interest (within the head and neck region) 2. SMOC: This involved the speech pathologist providing support to patients at their regional cancer site (i.e. patients’ local service) via email/telephone contact with their regional clinician as required	Health service costs (staff wages, equipment, and patient travel reimbursement), patient 1/carer costs (travel and wages), and patient-reported QoL	The new telepractice service showed a 12% average cost savings for the health service (*p* < 0.0058) and $40.05 saving per patient per referral, alongside a significant increase in QoL for both groups (*p* = 0.04)	1. Surveillance and management of psychosocial effects 2. Healthcare outcome: HRQoL 3. Implementation outcome: cost-effectiveness	High
Burns et al. [43], Australia	To examine service efficiency and participant satisfaction of the new telepractice service model	Two: 1. TMOC: an online consultation between the metro cancer centre and regional centres, utilising a videoconferencing unit with audiovisual recordings of the clinical area of interest (within the head and neck region) 2. SMOC: This involved the speech pathologist providing support to patients at their regional cancer site (i.e. patients’ local service) via email/telephone contact with their regional clinician as required	Primary outcome: efficiency of TMOC compared to SMOC Secondary outcomes: consumer and clinician satisfaction	TMOC provided a speech pathology service with greater efficiency than standard care. Both patients and consumers were satisfied	1. Surveillance and management of physical effects 2. Implementation outcome: satisfaction	High
Cascella et al. [36], Italy	To describe a single-centre experience with telemedicine for cancer pain management during and after the COVID-19 era	Single-arm. With two periods of treatment: during COVID-19 and after COVID 19	Dropout rate from the remote process, multiprofessional consultations, and distribution of visits on the national territory ECOG performance status	This telemedicine programme for cancer pain management offers valuable insights to establish effective and safe pathways for vulnerable populations	1. Surveillance and management of physical effects	Medium
DeGuzman et al. [39], USA	To assess the preliminary efficacy and fidelity of a nurse-led telemedicine intervention targeting rural cancer survivors’ distress symptoms	Single-arm quasi-experimental design	1. Cancer-related distress (DT) 2. Symptoms and unmet needs (SF-SUNS)	All domains demonstrated improvements post-intervention, with the largest in the emotional domain	1. Surveillance and management of psychosocial effects 2. Implementation outcome: fidelity	Low
Dirkse et al. [41], Canada	To compare the effectiveness and acceptability of two low-intensity methods of delivering iCBT for anxiety and depression symptoms in cancer survivors	Intervention: a ‘Wellbeing course’ designed to reduce maladaptive thoughts and behaviours linked to depression and anxiety while promoting healthy cognitive and behavioural patterns. The course spans 5 lessons to be completed within 8 weeks Two intervention conditions: 1. Self-guided—participants worked independently on this course 2. Technician guided—participants had weekly contact with a technician who summarised content, answered questions, reinforced progress, encouraged practice of skills, and normalised challenges of treatment	Primary outcomes: 1. Depression—(PHQ-9) Secondary outcomes: 1. (FCRI-SF) 2. QoL—Short Form Health Survey 3. Treatment satisfaction	1. Improvements in depression, anxiety, mental health-related QoL, and FCR. High satisfaction was reported	1. Surveillance and management of psychosocial effects 2. Healthcare outcome: HRQoL 3. Implementation outcome: satisfaction	High
Kang et al. [45], Australia	To evaluate the safety of administering immune checkpoint inhibitors and monitoring for immune-related adverse events using the Teleoncology model of care	Single-arm	Rates of immune-related adverse events and deaths (safety)	The results suggest that with adequate governance and clinical resources, immune checkpoint inhibitors can be administered safely using teleoncology models to rural and remote towns	Mortality outcome	Low
Kroenke et al. [33], USA	To assess the effectiveness of centralised telephone-based care management paired with automated symptom monitoring in alleviating depression and pain among cancer patients	Two-arm, randomised, receiving telephone-based intervention or receiving usual care	Depression (HSCL-20) BPI severity	Patients in the intervention group had greater improvements in BPI pain severity and depression over the 12 months of the trial than control	1. Surveillance and management of physical effects 2. Surveillance and management of psychosocial effects 3. Healthcare outcome: HRQoL	High
Peterson et al. [48], USA	To assess the feasibility and acceptability of remote data collection using software in three studies across diverse oncology settings. These studies focused on adherence to swallowing exercises in head and neck cancer patients during radiation therapy	Single-arm, study 2, smartphone with application for self-video capabilities and collection of patient-reported outcomes. HNC study participants used a study-provided smartphone to video-record all swallowing exercise sessions prescribed by their speech pathologist and, once-a-day, to self-initiate an assessment of symptoms and adherence to swallowing exercises	Capture adherence to swallowing exercises via video; collect treatment-related symptoms and self-report of swallowing exercises	Most HNC participants liked that device use provided motivation or a reminder to do the swallowing exercises and that using the devices was easy and convenient. A subset of HNC participants indicated that recording the videos took too much time and that the recordings were harder to do as treatment progressed	1. Surveillance and management of physical effects 2. Implementation outcome: satisfaction and feasibility	Low
Risendal et al. [49], USA	To assess the feasibility and acceptability of delivering the CTS programme, an adaptation of the evidence-based Chronic Disease Self-Management Programme (Stanford)	Single-arm. Six sessions as part of the CTS workshop, content included; restoration of self-confidence, adjustment to changed self, and confidence to self-manage cancer-related problems to promote successful coping and recovery of well-being following a cancer diagnosis	Participation and completion rates; satisfaction; evaluating content; recommendation; impact	95% of the participants were satisfied with the programme content and leaders and would recommend the programme to friends and family. A large number of caregivers attended (just over 14% of participants)	1. Client outcome: satisfaction 2. Implementation outcome: feasibility	Low
Sandell et al. [46], Australia	To evaluate patients’ acceptance of a shared cancer follow-up model of care between their general practitioner and radiation oncologist using the theoretical framework of acceptability, in the Illawarra Shoalhaven Local Health District	Single-arm	Acceptability	1. Majority (83%) of the participants accepted shared-care 2. No statistically significant results were found with patient acceptance of shared care between the tumour group	Implementation outcome: acceptability	Medium
Silva-Nash et al. [42], USA	To report the efficacy and safety of an advanced practice provider-led head and neck cancer survivorship clinic	NA, retrospective chart review, enrolment in a survivorship clinic	Efficacy; safety	Recurrence was detected in 50 patients (8.8%). 88.9% patients with locoregional recurrence were candidates for curative-intent salvage surgery	1. Prevention and surveillance for recurrence and new cancers	Low
Sivabalan and Upasani. [38], India	To assess the effectiveness of nursing interventions on physical and psychological outcome among cancer patients undergoing chemotherapy	Two-arm, randomised, receiving intervention or receiving routine care. Nursing interventions consisted of IV access care, oral care, back massage, progressive muscle relaxation therapy, breathing exercises, flexibility exercises, infection control measures, nutritional care, counselling, and spiritual care with pre-determined schedule, which were delivered with a pre-determined frequency throughout the course of hospital stay. Routine care consisted of IV access care, oral care, back massage, and infection control measures, and it was also delivered throughout a course of hospital stay	Physical outcome variables included chemotherapy symptoms, pain, fatigue, oral mucositis, nausea and vomiting, and extravasation Psychological outcome variables included emotional well-being, anxiety, depression, and patient concerns	Cancer patients who received nursing interventions had improved post-test mean scores on chemotherapy symptoms, pain and fatigue, and emotional well-being, anxiety and depression, than the patients who received routine care (*p* < 0.05). Nursing interventions were well accepted by cancer patients	1. Surveillance and management of physical effects 2. Surveillance and management of psychosocial effects 3. Implementation outcome: acceptability	Low
Yoo et al. [44], USA	To investigate the incremental cost-effectiveness of the INCPAD telecare management trial intervention	Two-arms, randomised, receiving the intervention or receiving usual care. The intervention was centralised telecare management coupled with automated symptom monitoring for patients with cancer	Cost-effectiveness	The intervention group reported an increase in depression free days and QoL in the intervention group as compared to usual care. The intervention was found to be cost-effective	1. Cost-effectiveness	High
Zernicke et al. [37], Canada	To assess the effects of participation in online MBCR on patient-reported outcomes in cancer patients	Single-arm, pre and post. The intervention consisted of 8 weekly 2-h online classes plus an online 6-h weekend retreat	POMS; CSOSI; FFMQ; FACIT-Sp; PTGI	Improvements in stress symptoms, spirituality (for younger participants), and PTG (in men) were observed. Participants were satisfied and would recommend the programme to other people living with cancer	1. Surveillance and management of physical effects 2. Surveillance and management of psychosocial effects 3. Client outcome: satisfaction	High

*BPI* Brief Pain Inventory, *CSOSI* Calgary Symptoms of Stress Inventory, *CTS* Cancer Thriving and Surviving, *DT* distress thermometer, *ECOG* Eastern Cooperative Oncology Group, *FACIT-Sp* Functional Assessment of Chronic Illness Therapy Spiritual Well-Being, *FCRI-SF* fear of cancer recurrence-short form, *FMY* finding my way, *FFMQ* Five Facet Mindfulness Questionnaire, *HSCL-20* Hopkins Symptom Checklist, *HRQoL* health-related QoL, *INCPAD* Indiana Cancer Pain and Depression, *iCBT* Internet-based cognitive behaviour therapy, *MBCR* mindfulness-based cancer recovery, *PHQ-9* Patient Health Ques, *PTGI* Post-Traumatic Growth Inventory, *POMS* Profile of Mood States, *QoL* quality of life, *SF-SUNS* Short Form Survivor Unmet Needs Survey, *SMOC* standard model of care, *TSUQ* Telemedicine Satisfaction and Usefulness Questionnaire, *TMOC* telepractice model of care

#### Domains of cancer survivorship pertaining to cancer and its treatment

1. Surveillance and management of physical effects
  Half of the included studies explored the effects of different models of cancer follow-up care on the physical effects of cancer and its treatment. These studies focused on addressing prominent physical symptoms and treatment-related effects including swallowing (*n* = 3), pain (*n* = 3), communication impairment (*n* = 2), and fatigue (*n* = 2). Less frequently reported outcomes (1 study each) included oral mucositis, nausea, and vomiting. Out of these, five care models improved outcomes such as communication issues, swallowing difficulties, pain, and fatigue. Kroenke et al. [33] found that patients enrolled in a nurse-led telehealth symptom management programme reported significant improvements in pain severity (primary outcome) as compared to participants in usual care even at 12 months of follow-up. Likewise, two models effectively utilised telehealth to remotely intervene and manage complex symptoms arising from laryngectomy such as communication and swallowing difficulties [34, 35]. Both of these models, led by speech pathologists, were effective in reducing the financial burden for rural patients by minimising travel expenses to speech pathologist services.
  Similarly, another telemedicine-based model was found to be effective in addressing cancer pain [36]. Two models demonstrated improvements in addressing chemotherapy-related symptoms such as fatigue, with one carried out remotely [37] and the other administered in-person [38]. However, this nurse-led intervention did not yield improvements in other symptoms such as oral mucositis, nausea, and vomiting [38]. Overall, these results indicate the effectiveness of various models of care in managing symptoms, with telehealth showing a particular promise in improving accessibility and reducing the financial burden for rural patients.
2. Surveillance and management of psychosocial effects
  Six studies addressed psychosocial effects, four of which had psychosocial outcomes as their primary outcome including cancer-related distress (*n* = 3), depression (*n* = 4), anxiety (*n* = 3), and quality of life (QoL) (*n* = 2). Less common psychosocial concerns addressed were emotional well-being, spiritual well-being, patient concern, mood, post-traumatic growth, and fear of cancer recurrence (FCR) (*n* = 1 each). Three interventions were delivered online, one was delivered via video, two administered via telehealth, and one in-person. Three of the interventions were nurse-led, while the remaining three were self-guided. Both telehealth interventions were effective in reducing cancer-related distress and depression [33, 39].
  Two interventions evaluated the effect of an evidence-based cognitive behaviour approach on outcomes such as depression, anxiety, FCR, and QoL [40, 41]. The ‘Finding My Way’ intervention [40] did not produce any between-group effects on the primary outcome (cancer-specific distress) nor secondary outcomes related to global quality of life. Nonetheless, the intervention improved emotional functioning and a decrease in health service utilisation among participants. Dirkse et al. [41] found large effect sizes on depression, anxiety, and mental health-related QoL (Cohen’s *d* ranging from 0.98 to 1.86) and moderate effects on FCR (Cohen’s *d*, 0.65–0.78). Similarly, another model of care based on mindfulness demonstrated greater improvements in stress symptoms and post-traumatic growth [37]. Importantly, both were self-guided and were delivered online [37, 41].
3. Prevention and surveillance for recurrence and new cancers
  One study reported on this domain [42]. The study evaluated the effectiveness of an advanced provider-led head and neck survivorship clinic. Results indicated that this model was well received and was acceptable among providers, with a detection rate of 10.7% for cancer recurrence and secondary primary cancers. Additionally, 12 new primary cases including HNC were discovered.

#### Healthcare outcomes

This was also categorised according to outcome measures identified by the Cancer Survivorship Care Quality Framework which includes health-related QoL/function, emergency services/hospitalisations, costs, and mortality. Three studies explored the effects of different models of care on health-related QoL [34, 41, 43]. Two studies reported significant improvements in QoL, with one study noting substantial within-group effect sizes on mental QoL post-treatment [41] and the second [43] also reporting significant improvement. The third study did not find any significant between-group (intervention vs control) effects on QoL in a nurse-led model of care [33]. Two studies assessed cost outcomes [43, 44]. Both care models were found to be cost-effective, with Burns et al. [43] reporting an average cost saving of AUD 40.05 per patient per referral. Only one study reported outcomes on mortality [45]. No studies explored outcomes on emergency services/hospitalisations.

### Effects of models of care on implementation-based conceptual framework of implementation outcomes

#### Implementation outcomes

Ten studies reported on implementation outcomes such as acceptability (*n* = 5), feasibility (*n* = 3), cost (*n* = 2), and fidelity (*n* = 1). Almost all interventions were acceptable among participants; however, two studies were rated low in quality and one medium [46]. For example, participants in a nurse-led intervention (administered in-person) expressed satisfaction with the programme [38], while those utilising remote technology found it easy to use and convenient [47, 48].

With regard to feasibility, all models of care were generally feasible as indicated by higher completion rates by participants across different time points. A study by Peterson et al. [48] reported a 97% adherence rate to remotely delivered swallowing exercises, while another study found that 84% of participants attended at least four out of six sessions [49].

Two studies reported on cost-effectiveness. One study demonstrated that the model (a telehealth care model primarily focused on cancer pain and depression) was effective in lowering healthcare costs [44], and in another study, a telehealth speech pathology service model demonstrated cost-effectiveness by reducing both health service and patient costs compared to standard care [43]. This study reported an average cost saving of AUD 59 per referral, AUD 16 in travel costs, and AUD 24 in time/wages for the patient.

Lastly, only one study addressed fidelity [39]. This was a telemedicine-delivered nursing intervention that targeted cancer-related distress. The programme was delivered as intended according to feedback from nurses.

#### Service outcomes

Only two studies reported service-related outcomes focusing on efficiency and safety (*n* = 1 each). Burns and colleagues [35] investigated the service efficiency of a telehealth model of care compared to usual care. Their findings indicated that the telehealth model led to greater efficiency, as evidenced by a reduction in the number and duration of contact events per referral. Notably, patients in the telehealth group experienced the added benefit of not needing to travel for their appointments, highlighting the convenience and accessibility afforded by this model.

#### Client outcomes

Across eight studies, satisfaction emerged as a prominent client outcome measure. The majority of participants across these studies expressed a high level of satisfaction with the various models of care administered. For instance, in a study by Risendal et al. [49], 65% of the respondents reported being ‘very satisfied’ with the intervention content. Participants in another intervention led by speech pathologists found the instructions easy to understand and expressed comfort in using technology [34]. Likewise, participants allocated to a telehealth model of care reported higher satisfaction levels than those receiving usual care [35]. Moreover, nearly all participants (98%) in a study by Dirkse et al. [41] expressed willingness to recommend internet-based psychotherapy to others and that completion of the intervention was worth their time. These findings align with Zernicke’s [37] study, where participants expressed satisfaction and indicated a willingness to recommend similar interventions to fellow cancer survivors.

## Discussion

While previous reviews have explored survivorship needs for people living with and beyond cancer, this is the first review to synthesise the evidence on models of care in addressing such needs for people in rural areas with HNC. We identified 14 studies, including eight randomised controlled trials assessing models of care for rural HNC patients, with half of these studies scoring high in quality ratings. It is worthwhile noting that the care models evaluated were based on ‘management’ outcomes rather than ‘surveillance’, as the framework proposes [31]. Majority of the included studies evaluated the models of care on physical and psychosocial effects of cancer treatment. However, no studies assessed the outcomes of models of care on managing chronic conditions and only two studies examined cost-related outcomes. Overall, the findings suggest that most of the care models are effective. Moreover, the review has identified potential areas of research in underrepresented domains and ways to improve the existing care models and develop new ones to address unique concerns reported by rural HNC survivors.

The predominant physical symptoms that were addressed included issues in swallowing, communication, and pain. These care models demonstrated success in managing the symptoms, with a considerable number being administered remotely. It is well known that rurality is associated with poor outcomes, such as reduced QoL and challenges in accessing healthcare [50, 51]. Access to care is a significant factor driving geographical disparities in cancer outcomes [52] and is linked to high unmet needs [24]. Overall, our data suggest that telehealth and internet-delivered interventions are well received and positively impact participant satisfaction levels. Remote telehealth models of care provide promising evidence for delivering routine survivorship care to low-risk patients by effectively reducing the travel burden [53]. However, recovery from cancer often presents various other significant impairments in physical functioning such as hearing loss, lymphoedema, or obstructive sleep apnoea [54]. None of the existing models of care targeted these issues. Additionally, due to the complex anatomy of the head and neck region, some physical issues such as difficulty in breathing require in-person management. Hence, the effectiveness of telehealth interventions for addressing these complex outcomes remains uncertain for rural patients, and satellite clinics may emerge as useful in this instance.

Most studies that evaluated psychological concerns yielded noteworthy results in effectively managing issues like depression, anxiety, and cancer-related distress [38–41]. However, several of these studies were rated low in quality with low retention rates. Nonetheless, these studies have important clinical implications as they were nurse-led and have demonstrated effectiveness in terms of addressing unmet emotional needs. However, only one study attempted to address FCR as a secondary outcome measure [41] and evaluated the effectiveness of iCBT (internet-delivered cognitive behavioural therapy) in managing FCR. Previous research has also indicated the effectiveness of evidence-based therapies such as CBT in addressing cancer-specific anxiety (or FCR) in patients with other cancer types [55]. In the literature, FCR is consistently reported as a topmost unmet need, and managing high levels of FCR has been identified as a research priority [56]. Hence, addressing FCR is critical in providing survivorship care, and future research should employ a large clinical trial with longer follow-up to demonstrate clinically significant benefits. Overall, findings show the effectiveness of online evidence-based interventions such as CBT, which is valuable for rural patients who have less access to mental health services and could be scalable. We also found that none of the care models targeted neurocognitive effects post-treatment and existential issues, such as fear of death, common in patients with advanced disease [57].

On the contrary, individuals with favourable prognoses are typically expected to manage their own needs relating to physical (such as pain), psychological (for example, anxiety), and other comorbidities [58]. This is consistent with the findings of the current review, which revealed that some care models were based on self-management practices. However, research has shown that rural cancer survivors are not always provided with sufficient information to self-manage their recovery [59]. For example, a review of 43 randomised controlled trials of digital interventions revealed that interventions guided by healthcare providers are more likely to promote engagement and result in significant improvements in psychosocial outcomes, such as anxiety and distress [60]. It becomes important to understand what self-management practices or health behaviours work well for rural survivors. Further studies are also necessary to investigate health resource utilisation in rural settings, including psychosocial support services, emergency hospitalisation, and mortality, as the framework suggests [31]. Therefore, addressing these concerns is critical, and future care models should be tailored to meet the needs of local rural communities [61].

Studies have shown that people living in rural areas have more health-related problems as compared to their urban counterparts [62]. In fact, a study by Palmer et al. [63] found that people living with cancer in rural areas often report physical and health promotion needs. However, it is important to note that no studies included in this review evaluated outcomes of general healthcare within the Cancer Survivorship Care Quality Framework [31]—that is, management of chronic medical conditions and health promotion/disease prevention were not addressed. It is known that a considerable proportion of patients with HNC endure chronic conditions such as hypertension and diabetes [64] and are at risk of developing another cancer (for example, lung cancer, due to ongoing smoking behaviours) [65]. The chronic conditions/comorbidities are influenced by socio-demographic factors like age and geographical location and are associated with higher mortality rates [25, 66]. For instance, geographical disparities in cancer-related outcomes have been attributed to differences in health-related behaviours [67] with rural residents reporting higher rates of alcohol intake and smoking compared to urban individuals [68]. Thus, models of care emphasising health promotion and lifestyle behaviour assessment could be instrumental in addressing this disparity [31].

The findings from the current review also suggest that the majority of models were led by nurses and speech pathologists. However, we did find one model of care that was led by GP. The involvement of GPs is crucial in the care of rural patients as the specialist services are often located further away. In addition to cancer surveillance, GPs play a vital role in managing comorbid illness, secondary prevention, health promotion, and care coordination [69]. Hence, developing a model of care led by GPs becomes important in the context of rural healthcare and sustainability. It is also worth noting that most of the current models of post-treatment care is primarily oncology specialist-led and tend to focus more on the surveillance of cancer and often fail to address other aspects of holistic care such as psychosocial and chronic conditions [69]. According to a study conducted in Canada, GPs are willing to accept exclusive follow-up care after completion of active treatment, but they may be hesitant for complex and high-risk patients [70] and may require additional training. Therefore, a high proportion of survivors are followed up by multiple providers [71], which highlights the need for effective care coordination between multiple health professionals. However, it is important to acknowledge that coordinated survivorship care is complex and can be resource-intensive requiring additional workforce and funding [72]. Hence, there is an urgent need for funders to allocate research funding to evaluate the effects of various models of survivorship care tailored to rural HNC survivors [73].

### Implications

This review demonstrates the effectiveness of various models of care in addressing the survivorship needs of rural HNC patients who face unique challenges, such as limited access to specialist healthcare services, transportation barriers, and socioeconomic disparities. These challenges must be taken into consideration when developing a model of care. As such, the implementation pathway of the included care models is not well established, with only a few studies evaluating the implementation outcomes. For example, cost-effectiveness, a crucial implementation outcome according to Proctor [32], was reported in only two studies [43, 44]. Moreover, none of the included studies evaluated ‘sustainability’ of these models within a service setting, indicating significant ‘research to practice gap’ [74]. Understanding sustainability is crucial for the successful delivery of new care models in community-based settings. Future research must extend beyond healthcare outcomes such as quality of life and execute robust economic evaluations to support the integration of care models into real-world settings. Therefore, there is a need for additional research, particularly in implementation science to improve access and promote equitable care, considering the unique needs of this patient group [12].

More recently, attention has been shifted towards the development of shared-care models. Such models entail integrating primary care and specialist care models in follow-up care [75, 76]. For instance, a systematic review of eight RCTs demonstrated the effectiveness of shared care in terms of QoL, mental health, addressing unmet needs, and clinical outcomes on par with usual care [76]. Additionally, two randomised trials investigating shared care in patients with colorectal and prostate cancers [15, 16] concluded that patients strongly preferred the shared care model over usual care and found no significant differences in QoL outcomes between groups. It was also concluded that shared care models were cost-effective for the healthcare system as compared to the usual oncology-led models. Therefore, similar trials could be conducted for individuals with HNC, which are cost-effective, although only two studies adopting shared care were identified. However, implementation of shared care requires careful planning in rural settings [77].

### Strengths and limitations

The current review provides valuable insight into the models of care available for rural HNC survivors. Strengths of the review include its broad inclusion criteria and diverse settings in which the models were implemented. Additionally, the majority of the included studies were assessed as having high or medium methodological quality. However, there are some limitations to the review that should be acknowledged. Despite the broad inclusion criteria, the search yielded a relatively small number of articles. Also, some studies included participants with different types of cancer, and only a few had HNC, potentially limiting the applicability of the findings to HNC patients specifically. Studies were also excluded if they did not report outcomes for HNC participants or if the number of HNC participants was not available. Additionally, studies were excluded if the authors did not provide sufficient information on whether participants were recruited from rural areas. It is important to note that the definition of rurality varies across countries, which may have resulted in the exclusion of informative studies.

## Conclusion

This review revealed that the majority of available care models were delivered by nurses and allied healthcare professionals, particularly speech pathologists, and were often administered through online or telehealth platforms. Given the unique physical and psychosocial needs of rural HNC patients, compounded by access issues, further evidence is required to determine the efficacy of self-guided care models with reduced input from healthcare teams. It was also observed that most of the included care models are not extensively implemented in institutional settings, highlighting the need for additional research to assess implementation outcomes, particularly cost-effectiveness and long-term sustainability. The review also suggests that shared care models may be more practical for patients requiring long-term follow-up, emphasising the importance of general practice closer to home. However, challenges related to workforce availability and funding allocation may impede the widespread adoption of such models. In conclusion, future care models should prioritise accessibility, affordability, and patient-centeredness to optimise outcomes for individuals with HNC residing in rural areas.

## Supplementary information

Below is the link to the electronic supplementary material.

Supplementary file1 (DOCX 27 KB)

Supplementary file2 (DOCX 37 KB)

## Data Availability

The data that support this manuscript are available from the researchers upon reasonable request.
