# Supplementary material for: Models of survivorship care in patients with head and neck cancer in regional, rural, and remote areas: a systematic review
Source: J Cancer Surviv. 2024 Jul 20;20(1):163–80. doi: 10.1007/s11764-024-01643-x (PMC12906516; doi:10.1007/s11764-024-01643-x)
Supplement: Supplementary file 1 — Supplementary file1 (DOCX 27 KB) [file 11764_2024_1643_MOESM1_ESM.docx]

1. **PubMed:**

((((((cancer*) OR (oncology)) OR (neoplasm*)) OR (carcinoma)) OR (tumor*)) OR (tumour*)) AND ((((((((((((((((((((((((((((((((((mouth neoplasms) OR (gingival neoplasms)) OR (tongue neoplasms)) OR (oral)) OR (mouth)) OR (head)) OR (neck)) OR (pharynx)) OR (pharyngeal)) OR (nasopharynx)) OR (nasopharyngeal)) OR (aerodigestive)) OR (larynx)) OR (laryngeal*)) OR (face)) OR (facial)) OR (tongue*)) OR (lip*)) OR (hypopharyn*)) OR (buccal)) OR (tonsil*)) OR (vocal cord*)) OR (gingival)) OR (leukoplakia)) OR (palatal)) OR (parotid)) OR (sublingual)) OR (otorhinolaryn*)) OR (submandibular)) OR (ear)) OR (nose)) OR (maxillary sinus)) OR (orophar*)) OR (tracheal)) AND (((regional) OR (remote)) OR (rural)) AND (((((((((((((((Model* of care) OR (Survivorship care plan)) OR (follow up care)) OR (nurse-led)) OR (shared care)) OR (primary care)) OR (provider-led)) OR (General Practitioner-led)) OR (oncology-led)) OR (end of treatment)) OR (psycho* care)) OR (educational intervention)) OR (post treatment)) OR (palliative care)) OR (end of life care))

1. **Scopus:**

( TITLE-ABS-KEY ( cancer* OR oncology OR neoplasm* OR carcinoma OR tumor* OR tumour* ) AND TITLE-ABS-KEY ( "mouth neoplasms" OR "gingival neoplasms" OR "tongue neoplasms" OR oral OR mouth OR head OR neck OR pharynx OR pharyngeal OR nasopharynx OR nasopharyngeal OR aerodigestive OR larynx OR laryngeal* OR face OR facial OR tongue* OR lip* OR hypopharyn* OR buccal OR tonsil* OR vocal OR cord* OR gingival OR leukoplakia OR palatal OR parotid OR sublingual OR otorhinolaryn* OR submandibular OR ear OR nose OR "maxillary sinus" OR orophar* OR tracheal ) AND TITLE-ABS-KEY ( regional OR remote OR rural ) AND TITLE-ABS-KEY ( "model* of care" OR "survivorship care plan" OR "follow up care" OR "nurse-led" OR "shared care" OR "primary care" OR "provider-led" OR "general practitioner-led" OR "oncology-led" OR "end of treatment" OR "psycho* care" OR "educational intervention" OR "post treatment" OR "palliative care" OR "end of life care" ) )

1. **Web of Science:**

(((ALL=("Cancer*" OR "Oncology" OR "Neoplasm*" OR "Carcinoma" OR "Tumor*" OR "Tumour*")) AND ALL=("Mouth Neoplasms" OR "Gingival Neoplasms" OR "tongue Neoplasms" OR "oral" OR "mouth" OR "head" OR "neck" OR "pharynx" OR "pharyngeal" OR "nasopharynx" OR "nasopharyngeal" OR "aerodigestive" OR "larynx" OR "laryngeal*" OR "face" OR "facial" OR "tongue*" OR "lip*" OR "hypopharyn*" OR "buccal" OR "tonsil*" OR "vocal cord*" OR "gingival" OR "leukoplakia" OR "palatal" OR "parotid" OR "sublingual" OR "otorhinolaryn*" OR "submandibular" OR "ear" OR "nose" OR "maxillary sinus" OR "orophar*" OR "tracheal")) AND ALL=("Regional" OR "Remote" OR "Rural")) AND ALL=("Model* of care" OR "Survivorship care plan" OR "Follow up care" OR "Nurse-led" OR "Shared care" OR "Primary care" OR "Provider-led" OR "General Practitioner-led" OR "Oncology-led" OR "End of treatment" OR "Psycho* care" OR "Educational intervention" OR "Post treatment" OR "Palliative care" OR "End of life care")

1. **PsycINFO:**

1 cancer.mp.

2 oncology.mp or exp Oncology/

3 neoplasm.mp or exp Neoplasms/

4 carcinoma.mp or exp Neoplasms/

5 tumor.mp or exp Neoplasms/

6 tumour.mp

7 Mouth Neoplasms.mp

8 Gingival Neoplasm.mp [mp=title, abstract, heading word, table of contents, key concepts, original title, tests, & measures, mesh word]

9 tongue neoplasms.mp

10 oral.mp

11 mouth.mp

12 head.mp

13 exp “Neck (Anatomy)”/ or neck.mp.

14 pharynx.mp or exp Pharynx /

15 pharyngeal.mp

16 exp Neoplasms/ or exp Pharynx / or nasopharynx.mp

17 exp Neoplasms/ or aerodigestive.mp.

18 larynx.mp or exp Larynx/

19 exp Neoplasms/ or laryngeal.mp

20 face.mp or exp “Face (Anatomy)”/

21 facial.mp

22 exp Tongue/ or tongue.mp.

23 exp “Lips (Face)”/ or lip.mp.

24 exp Neoplasms/ or hypopharynx.mp.

25 exp Neoplasms/ or hypopharyngeal.mp.

26 exp Neoplasms/ or buccal.mp.

27 exp Neoplasms/ or tonsil.mp.

28 vocal cord.mp. or exp Vocal Cords/

29 gingival.mp.

30 exp Neoplasms/ or leukoplakia.mp.

31 palatal.mp.

32 exp Neoplasms/ or parotid.mp.

33 sublingual.mp.

34 otorhinolaryngeal.mp.

35 submandibular.mp.

36 ear.mp or exp “Ear (Anatomy)”/

37 nose.mp. or exp Nose/

38 exp Neoplasms/ or maxillary sinus.mp.

39 exp Neoplasms/ or oropharynx.mp.

40 exp Neoplasms/ or oropharyngeal.mp.

41 tracheal.mp.

42 exp Geography/ or regional.mp.

43 exp Indigenous Populations/ or exp Rural Environments/ or remote.mp.

44 exp Rural Health/or exp Rural Environments/ or rural.mp.

45 exp Health Care Services/or models of care.mp.

46 exp Neoplasms/ or survivorship care plan.mp.

47 exp Neoplasms/ or exp Survivors/ or Follow up care.mp.

48 exp Neoplasms/ or exp Health Care Services/ or exp Intervention/ or Nurse-led.mp.

49 exp General Practitioners/ or exp Neoplasms/ or Shared care.mp.

50 Primary Care.mp. or exp Primary Health Care/

51 Provider-led.mp.

52 exp General Practitioners/ or exp Primary Health Care/ or General Practitioner-led.mp.

53 exp Neoplasms/ or exp Health Care Services/ or oncology led.mp.

54 exp Neoplasms/ or end of treatment

55 exp Neoplasms/ or exp Survivors/ or psychosocial care.mp. or exp Mental Health Services/

56 educational intervention.mp.

57 exp Survivors/ or exp Neoplasms/ or post treatment.mp.

58 palliative care.mp. or exp Palliative Care/

1. end of life care.mp. or exp Palliative Care/
2. **Embase:**

1 cancer.mp.

2 oncology.mp or oncology/

3 neoplasm/ or neoplasms*.mp.

4 “head and neck carcinoma”/ or carcinoma.mp. or tongue carcinoma/ or nasopharynx carcinoma/ or “head and neck carcinoma squamous cell carcinoma cell line”/ or “head and neck squamous cell carcinoma”/ or oropharynx carcinoma/ or larynx carcinoma/ or parotid gland carcinoma/ or lip carcinoma/ or carcinoma/ or mouth carcinoma/ or tonsil carcinoma/ or nose carcinoma/ or maxilla sinus carcinoma/ or salivary gland carcinoma/

5 tumor.mp or neoplasm/

6 tumour.mp or neoplasm/

7 Mouth Neoplasms.mp. or mouth tumor/

8 Gingival Neoplasm. or gingiva tumor/

9 tongue neoplasms.mp. or tongue tumor/

10 oral.mp.

11 mouth/ or mouth tumor/ or mouth.mp. or mouth cancer/ or mouth carcinoma/ or mouth squamous cell carcinoma

12 head.mp. or “head and neck carcinoma”/ or “head and neck cancer”/ or “head and neck tumor”/ or head tumor/ or head

13 “head and neck carcinoma squamous cell carcinoma cell line”/ or neck cancer/ or “head and neck metastasis”/or “head and neck carcinoma”/ or neck.mp. or “head and neck cancer”/ or head and neck carcinoma squamous cell carcinoma”/ or neck/ or “head and neck cell line”/

14 pharynx cancer or pharynx/ or pharynx tumor/ or pharynx.mp or pharynx carcinoma

15 pharyngeal.mp.

16 nasopharynx tumor/ or nasopharyngeal.mp. or nasopharynx/

17 aerodigestive.mp. or larynx cancer/

18 larynx cancer or larynx.mp or larynx/ or larynx tumor/ or larynx carcinoma/ or larynx squamous cell carcinoma

19 laryngeal*.mp

20 face.mp or exp/ or face cancer/

21 facial.mp.

22 tongue.mp. or tongue cancer/ or tongue squamous cell carcinoma cell line/ or tongue/ or tongue tumor/ or tongue cancer cell line/

23 lip cancer/or lip/ or lip carcinoma/or lip*.mp. or lip tumor

24 “head and neck cancer”/ or hypopharyn*.mp. or hypopharynx tumor/or hypopharynx carcinoma

25 buccal.mp.

26 tonsil tumor/ or tonsil/ or tonsil carcinoma/ or tonsil*.mp. or tonsil cancer/

27 vocal cord*.mp.

28 gingival.mp.

29 leukoplakia.mp. or leukoplakia/

30 palatal.mp.

31 parotid.mp. or parotid gland tumor/or parotid gland carcinoma/ or parotid gland cancer

32 sublingual.mp.

33 otorhinolaryn*.mp.

34 submandibular.mp.

35 ear.mp. or middle ear tumor/ or ear/or ear tumor/or ear cancer

36 nose tumor/ or nose/ or nose cancer or nose.mp. or nose cavity tumor/ or nose carcinoma/nose cavity cancer/

37 maxillary sinus.mp. or maxillary sinus/

38 oropharynx cancer/or orophar*.mp.

39 tracheal.mp.

40 regional.mp.

41 remote.mp.

42 rural.mp. or rural population/ or rural area/

43 survivorship/ or Survivorship need*.mp. or neoplasm/

44 model* of care.mp.

45 cancer survival/ or survivorship/ or neoplasm/ or survivorship care plan.mp. or cancer survivor/

46 Follow up care.mp.

47 nurse-led.mp.

48 Shared care.mp.

49 primary care.mp. or exp primary medical care/

50 Provider-led.mp.

51 exp General Practitioner/ or General Practitioner-led.mp.

52 or oncology-led.mp.

53 end of treatment.mp.

54 psychosocial care/ or psycho* care.mp.

55 educational intervention.mp.

56 post treatment.mp.

57 palliative care.mp.

58 end of life care.mp. or exp terminal care/

1. **Medline:**

1 cancer.mp.

2 oncology.mp.

3 neoplasm*.mp

4 “squamous cell carcinoma of head and neck”/or Carcinoma/ or carcinoma.mp.

5 tumor*.mp.

6 Neoplasms/ or tumour*.mp

7 Mouth Neoplasms.mp or Mouth Neoplasms/

8 Gingival Neoplasms.mp or Gingival Neoplasms/

9 tongue neoplasms.mp or Tongue Neoplasms/

10 oral.mp.

11 Mouth/ or mouth.mp

12 “squamous cell carcinoma of head and neck”/or Head/ or head.mp

13 Neck/ or neck.mp.

14 pharynx.mp or Pharynx /

15 pharyngeal.mp. or pharyngeal neoplasms/

16 nasopharynx.mp. or nasopharynx/

17 Nasopharyngeal Neoplasms/ or Nasopharyngeal Carcinoma/ or Nasopharyngeal.mp.

18 aerodigestive.mp.

19 Larynx/ or larynx.mp.

20 Laryngeal Neoplasm/ or laryngeal*.mp.

21 facial.mp.

22 face.mp. or Face/

23 Tongue Neoplasms/ or tongue/ or tongue*.mp.

23 Lip/ or lip*.mp. or Lip Neoplasms

24 Hypopharygeal Neoplasms/ orHypopharynx/or hypopharynx*.mp.

25 Mouth Neoplasms/ or buccal.mp.

26 tonsil*.mp.

27 vocal cord*.mp.

28 gingival.mp.

29 leukoplakia.mp. or Leukoplakia/

30 palatal.mp.

31 Parotid Neoplasms/ or parotid.mp.

32 sublingual.mp.

33 otorhinolaryn*.mp. or Otorhinolaryngologic Neoplasms/

34 Ear Neoplasms/ or ear.mp. or Ear/

35 tracheal.mp.

36 regional.mp.

37 remote.mp.

38 rural.mp. or Rural Population/

39 model* of care.mp.

40 Cancer Survivors/ or Survivorship/ or Survivorship Care Plan.mp.

41 nurse-led.mp.

42 shared care.mp.

43 primary care.mp. or exp Primary Health Care/

44 provider-led.mp. or “Delivery of Health Care”/

45 General Practitioners/ or General Practitioner-led.mp.

46 oncology-led.mp.

47 end of treatment.mp.

48 Provider-led.mp.

49 psycho* care.mp.

50 Patient Education as Topic/ or educational intervention.mp. or Health Education/

51 post treatment.mp.

52 Palliative Care.mp. or Palliative Care.mp.

53 end of life care.mp. or Terminal Care/

1. **CINAHL:**

S1 "cancer*"

S2 "oncology"

S3 "neoplasm*"

S4 "carcinoma"

S5 "tumor*"

S6 "tumour*"

S7 "Mouth Neoplasms"

S8 "Gingival Neoplasms"

S9 "tongue neoplasms"

S10 "oral"

S11 "mouth" OR (MH "Mouth Neoplasms+")

S12 "head" OR (MH "Head and Neck Neoplasms+")

S13 "neck"

S14 "pharynx" OR (MH "Pharyngeal Neoplasms+")

S15 "pharyngeal"

S16 "nasopharynx" OR (MH "Nasopharyngeal Carcinoma") OR (MH "Nasopharyngeal Neoplasms+")

S17 (MH "Nasopharyngeal Neoplasms+") OR (MH "Nasopharyngeal Carcinoma") OR "nasopharyngeal"

S18 "aerodigestive"

S19 "larynx"

S20 "laryngeal*" OR (MH "Laryngeal Neoplasms")

S21 "face"

S22 "facial" OR (MH "Facial Neoplasms+")

S23 "tongue*" OR (MH "Tongue Neoplasms")

S24 (MH "Lip Neoplasms") OR "lip*"

S25 (MH "Hypopharyngeal Neoplasms") OR "hypopharyn*"

S26 "buccal"

S27 "tonsil*" OR (MH "Tonsillar Neoplasms")

S28 "vocal cord*"

S29 (MH "Gingival Neoplasms") OR "gingival"

S30 "leukoplakia"

S31 (MH "Palatal Neoplasms") OR "palatal"

S32 (MH "Parotid Neoplasms") OR "parotid"

S33 "sublingual"

S34 "otorhinolaryn*" OR (MH "Otorhinolaryngologic Neoplasms+")

S35 "submandibular"

S36 (MH "Ear Neoplasms") OR "ear"

S37 (MH "Nose Neoplasms+") OR "nose"

S38 "maxillary sinus" OR (MH "Maxillary Neoplasms") OR (MH "Paranasal Sinus Neoplasms")

S39 "orophar*" OR (MH "Oropharyngeal Neoplasms+") OR (MH "Oropharynx+")

S40 "tracheal"

S41 "regional" OR (MH "Geographic Factors+") OR (MH "Regional Centers")

S42 (MH "Services for Australian Rural and Remote Allied Health") OR "remote" OR (MH "Rural Health Services") OR (MH "Remote Consultation")

S43 (MH "Rural Health Centers") OR "rural" OR (MH "Rural Areas") OR (MH "Rural Health Services") OR (MH "Rural Population") OR (MH "Hospitals, Rural") OR (MH "Rural Health") OR (MH "Services for Australian Rural and Remote Allied Health")

S44 "Survivorship need*" OR (MH "Survivorship")

S45 "unmet need*" OR (MH "Health Services Needs and Demand+")

S46 "Supportive care need*" OR (MH "Support, Psychosocial+")

S47 "Psycho* need*"

S48 "physical need*"

S49 "Social need*"

S50 "Spiritual need*"

S51 "Information* need*" OR (MH "Information Needs")

S52 "integrative care"

S53 "Model* of care"

S54 "survivorship care plan"

S55 "follow up care" OR (MH "Cancer Care Facilities")

S56 "Nurse-led"

S57 "shared care" OR (MH "Cancer Care Facilities") OR (MH "Shared Services, Health Care")

S58 "primary care" OR (MH "Primary Health Care")

S59 "Provider-led"

S60 "General Practitioner-led"

S61 "Oncology-led" OR (MH "Oncologic Care+") OR (MH "Oncologic Nursing+")

S62 "end of treatment"

S63 "Psycho* care"

S64 "educational intervention"

S65 "post treatment"

S66 (MH "Palliative Care") OR "palliative care" OR (MH "Cancer Care Facilities") OR (MH "Hospice and Palliative Nursing")

S67 "end of life care"

S68 S1 OR S2 OR S3 OR S4 OR S5 OR S6

S69 S7 OR S8 OR S9 OR S10 OR S11 OR S12 OR S13 OR S14 OR S15 OR S16 OR S17 OR S18 OR S19 OR S20 OR S21 OR S22 OR S23 OR S24 OR S25 OR S26 OR S27 OR S28 OR S29 OR S30 OR S31 OR S32 OR S33 OR S34 OR S35 OR S36 OR S37 OR S38 OR S39 OR S40

S70 S41 OR S42 OR S43

S71 S44 OR S45 OR S46 OR S47 OR S48 OR S49 OR S50 OR S51 OR S52

S72 S53 OR S54 OR S55 OR S56 OR S57 OR S58 OR S59 OR S60 OR S61 OR S62 OR S63 OR S64 OR S65 OR S66 OR S67

S73 S68 AND S69 AND S70 AND S71

S74 S68 AND S69 AND S70 AND S72
