# Supplementary material for: Models of survivorship care in patients with head and neck cancer in regional, rural, and remote areas: a systematic review
Source: J Cancer Surviv. 2024 Jul 20;20(1):163–80. doi: 10.1007/s11764-024-01643-x (PMC12906516; doi:10.1007/s11764-024-01643-x)
Supplement: Supplementary file 2 — Supplementary file2 (DOCX 37 KB) [file 11764_2024_1643_MOESM2_ESM.docx]

**Supplementary File**

***Methodology***

The appropriate JBI Critical Appraisal Tool was used for each study. Each item on the checklist was scored as follows:

- Yes (2)
- N/A (1)
- No or unclear (0)

The % total score was calculated as the sum of the scores divided by the maximum total score possible.

Example: For Non-randomised experimental studies, the maximum possible score is 18 points *(2 points/item * 9 items)*. DeGuzman (2022b) scored 16 points – therefore 16/18 = 98%.

Quality was assessed as:

- High (≥80%)
- Medium (60-79%)
- Low (<60%)

**Table 1** Risk of bias assessment for randomised controlled trials

| **Author (Year)** | Beatty (2019) | Burns (2017a) | Burns (2017b) | Dirkse (2020) | Kroenke (2010) | Sivabalan (2016) | Yoo (2014) | Zernicke (2016) |
| --- | --- | --- | --- | --- | --- | --- | --- | --- |
| Was true randomization used for assignment of participants to treatment groups? | Yes | Yes | Yes | Yes | Yes | No | Yes | Yes |
| Was allocation to treatment groups concealed? | Yes | Yes | Yes | Yes | Yes | No | Yes | Yes |
| Were treatment groups similar at the baseline? | Yes | Yes | Yes | Yes | Yes | Yes | Yes | Yes |
| Were participants blind to treatment assignment? | Yes | No | No | No | Yes | No | Yes | No |
| Were those delivering the treatment blind to treatment assignment? | Yes | No | No | No | Unclear | No | Unclear | No |
| Were treatment groups treated identically other than the intervention of interest? | Yes | Yes | Yes | Yes | Yes | Unclear | Yes | Yes |
| Were outcome assessors blind to treatment assignment? | N/A | N/A | N/A | N/A | N/A | N/A | N/A | N/A |
| Were outcomes measured in the same way for treatment groups? | Yes | Yes | Yes | Yes | Yes | Yes | Yes | Yes |
| Were outcomes measured in a reliable way? | Yes | Yes | Yes | Yes | Yes | Yes | Yes | Yes |
| Was follow up complete and if not, were differences between groups in terms of their follow up adequately described and analysed? | Yes | Yes | Yes | Yes | Yes | Yes | Yes | Yes |
| Were participants analysed in the groups to which they were randomized? | Yes | Yes | Yes | Yes | Yes | Yes | Yes | Yes |
| Was appropriate statistical analysis used? | Yes | Yes | Yes | Yes | Yes | Yes | Yes | Yes |
| Was the trial design appropriate and any deviations from the standard RCT design (individual randomization, parallel groups) accounted for in the conduct and analysis of the trial? | Yes | Yes | Yes | Yes | Yes | No | Yes | Yes |
| **Quality assessment** | ***96% (high)*** | ***81% (high)*** | ***81% (high)*** | ***81% (high)*** | ***88% (high)*** | ***50% (low)*** | ***88% (high)*** | ***81% (high)*** |

**Table 2** Risk of bias assessment for quasi-experimental studies

| **Author (Year)** | DeGuzman (2022) | Peterson (2021) | Risendal (2014) | Bernacchi (2023) |
| --- | --- | --- | --- | --- |
| Is it clear in the study what is the ‘cause’ and what is the ‘effect’ (i.e. there is no confusion about which variable comes first)? | Yes | Yes | Yes | Yes |
| Were the participants included in any comparisons similar? | No | N/A | N/A | N/A |
| Were the participants included in any comparisons receiving similar treatment/care, other than the exposure or intervention of interest? | Yes | N/A | N/A | N/A |
| Was there a control group? | No | No | No | No |
| Were there multiple measurements of the outcome both pre and post the intervention/exposure? | No | No | No | No |
| Was follow up complete and if not, were differences between groups in terms of their follow up adequately described and analyzed? | No | No | No | No |
| Were the outcomes of participants included in any comparisons measured in the same way? | Yes | N/A | N/A | Yes |
| Were outcomes measured in a reliable way? | Yes | Yes | No | Yes |
| Was appropriate statistical analysis used? | Yes | N/A | N/A | Yes |
| **Quality assessment** | ***56% (low)*** | ***44% (low)*** | ***33% (low)*** | ***56% (low)*** |

**Table 3** Risk of bias for cohort studies

| **Author (Year)** | Cascella (2023) | Silva-Nash (2022) | Kang (2023) |
| --- | --- | --- | --- |
| Were the two groups similar and recruited from the same population? | Yes | N/A | No |
| Were the exposures measured similarly to assign people to both exposed and unexposed groups? | Yes | N/A | N/A |
| Was the exposure measured in a valid and reliable way? | Yes | Yes | Yes |
| Were confounding factors identified? | No | No | No |
| Were strategies to deal with confounding factors stated? | No | No | No |
| Were the groups/participants free of the outcome at the start of the study (or at the moment of exposure)? | N/A | Yes | No |
| Were the outcomes measured in a valid and reliable way? | N/A | Yes | Yes |
| Was the follow up time reported and sufficient to be long enough for outcomes to occur? | N/A | Yes | N/A |
| Was follow up complete, and if not, were the reasons to loss to follow up described and explored? | Yes | Yes | N/A |
| Were strategies to address incomplete follow up utilised? | Yes | No | N/A |
| Was appropriate statistical analysis used? | Yes | N/A | Yes |
| ***Quality assessment*** | ***68% (medium)*** | ***59% (low)*** | ***56% (low)*** |

**Table 4** Risk of bias assessment for case series

| **Author (Year)** | Burns (2012) |
| --- | --- |
| Were there clear criteria for inclusion in the case series? | Yes |
| Was the condition measured in a standard, reliable way for all participants included in the case series? | Yes |
| Were valid methods used for identification of the condition for all participants included in the case series? | Yes |
| Did the case series have consecutive inclusion of participants? | Unclear |
| Did the case series have complete inclusion of participants? | Unclear |
| Was there clear reporting of the demographics of the participants in the study? | Yes |
| Was there clear reporting of clinical information of the participants? | Yes |
| Were the outcomes or follow up results of cases clearly reported? | Yes |
| Was there clear reporting of the presenting site(s)/clinic(s) demographic information? | Yes |
| Was statistical analysis appropriate? | N/A |
| **Quality assessment** | ***75% (medium)*** |

**Table 5** Risk of bias for analytical cross-sectional study

| **Author (Year)** | Sandell (2023) |
| --- | --- |
| Were the criteria for inclusion in the sample clearly defined? | Yes |
| Were the study subjects and the setting described in detail? | Yes |
| Was the exposure measured in a valid and reliable way? | Yes |
| Were objective, standard criteria used for measurement of the condition? | Yes |
| Were confounding factors identified? | No |
| Were strategies to deal with confounding factors stated? | No |
| Were the outcomes measured in a valid and reliable way? | Yes |
| Was appropriate statistical analysis used? | Yes |
| ***Quality assessment*** | ***75% (medium)*** |
